# Ethyl 3-[1-(2-hydroxy­phen­yl)eth­ylidene]carbazate

**DOI:** 10.1107/S1600536809044602

**Published:** 2009-10-31

**Authors:** Yu-Feng Li, Hai-Xing Liu, Fang-Fang Jian

**Affiliations:** a Microscale Science Institute, Department of Chemistry and Chemical Engineering, Weifang University, Weifang 261061, People’s Republic of China; b Microscale Science Institute, Weifang University, Weifang 261061, People’s Republic of China

## Abstract

The title compound, C_11_H_14_N_2_O_3_, was prepared by the reaction of ethyl carbazate and 1-(2-hydroxy­phen­yl)ethanone. In the crystal structure, mol­ecules are linked by inter­molecular N—H⋯O hydrogen bonds, forming centrosymmetric dimers. An intra­molecular O—H⋯N inter­action also occurs.

## Related literature

For the applications of Schiff base compounds, see: Cimerman *et al.* (1997 ▶); Forthe C=N double-bond length in a related structure, see: Girgis (2006 ▶).
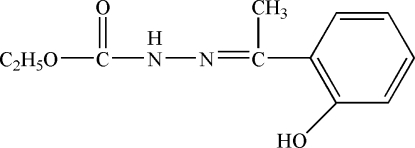


## Experimental

### 

#### Crystal data

C_11_H_14_N_2_O_3_
*M* *_r_* = 222.24
Triclinic, 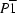

*a* = 5.4830 (11) Å
*b* = 10.191 (2) Å
*c* = 11.410 (2) Å
α = 112.53 (3)°
β = 95.79 (3)°
γ = 99.68 (3)°
*V* = 570.9 (2) Å^3^
*Z* = 2
Mo *K*α radiation
μ = 0.10 mm^−1^
*T* = 293 K
0.22 × 0.20 × 0.18 mm

#### Data collection

Bruker SMART CCD diffractometer
Absorption correction: multi-scan (*SADABS*; Sheldrick, 1996 ▶) *T*_min_ = 0.491, *T*_max_ = 0.728
5650 measured reflections
2597 independent reflections
1839 reflections with *I* > 2σ(*I*)
*R*_int_ = 0.016

#### Refinement

*R*[*F* ^2^ > 2σ(*F* ^2^)] = 0.047
*wR*(*F* ^2^) = 0.180
*S* = 1.08
2597 reflections
166 parameters
H atoms treated by a mixture of independent and constrained refinement
Δρ_max_ = 0.23 e Å^−3^
Δρ_min_ = −0.23 e Å^−3^

### 

Data collection: *SMART* (Bruker, 1997 ▶); cell refinement: *SAINT* (Bruker, 1997 ▶); data reduction: *SAINT*; program(s) used to solve structure: *SHELXS97* (Sheldrick, 2008 ▶); program(s) used to refine structure: *SHELXL97* (Sheldrick, 2008 ▶); molecular graphics: *SHELXTL* (Sheldrick, 2008 ▶); software used to prepare material for publication: *SHELXTL*.

## Supplementary Material

Crystal structure: contains datablocks global, I. DOI: 10.1107/S1600536809044602/lh2935sup1.cif

Structure factors: contains datablocks I. DOI: 10.1107/S1600536809044602/lh2935Isup2.hkl

Additional supplementary materials:  crystallographic information; 3D view; checkCIF report

## Figures and Tables

**Table 1:** Hydrogen-bond geometry (Å, °)

*D*—H⋯*A*	*D*—H	H⋯*A*	*D*⋯*A*	*D*—H⋯*A*
N2—H2*A*⋯O1^i^	0.92 (2)	2.05 (2)	2.9649 (19)	170.4 (16)
O3—H3*A*⋯N1	0.98 (3)	1.68 (3)	2.5728 (19)	149 (2)

Symmetry code: (i) 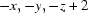.

**Fig. 1.**
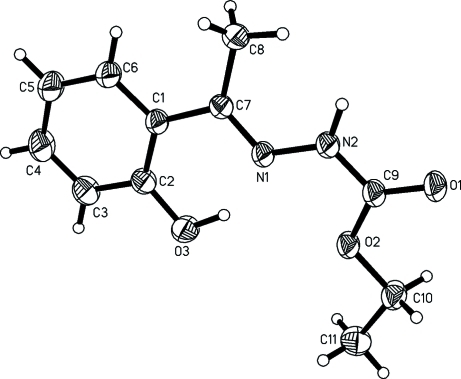
The molecular structure of (I) showing 30% probability displacement ellipsoids and the atom-numbering scheme.

## supplementary crystallographic information

### Comment

Schiff bases have received considerable attention in the literature and have potential analytical applications (Cimerman *et al.*, 1997). As part of our search for new schiff base compounds we synthesized the title compound (I), and its crystal structure is determined herein.

The molecular structure of (I) is shown in Fig. 1. The C7—N1 bond length of 1.2836 (18)Å is comparable with C—N double bond [1.281 (2) Å] reported (Girgis, 2006). In the crystal structure, molecules are linked by intermolecular N—H···O hydrogen bonds to form centrosymmetric dimers.

### Experimental

A mixture of the 1-(2-hydroxyphenyl)ethanone (0.1 mol), and Ethyl carbazate (0.1 mol) was stirred in refluxing ethanol (20 mL) for 4 h to afford the title compound (0.082 mol, yield 82%). Single crystals suitable for X-ray measurements were obtained by recrystallization of (I) from ethanol at room temperature.

### Refinement

H atoms bonded to the O atom, the N atom and those bonded to C8 were refined independently with isotropic displacement parameters. All other H atoms were fixed geometrically and allowed to ride on their attached atoms, with C—H distances = 0.93–0.97 Å *U*_iso_(H) = 1.2*U*_eq_(C) or 1.5*U*_eq_(C_methyl_).

### Crystal data

**Table:**

C_11_H_14_N_2_O_3_	*Z* = 2
*M**_r_* = 222.24	*F*(000) = 236
Triclinic, *P*1	*D*_x_ = 1.293 Mg m^−^^3^
Hall symbol: -P 1	Mo *K*α radiation, λ = 0.71073 Å
*a* = 5.4830 (11) Å	Cell parameters from 1974 reflections
*b* = 10.191 (2) Å	θ = 3.5–27.5°
*c* = 11.410 (2) Å	µ = 0.10 mm^−^^1^
α = 112.53 (3)°	*T* = 293 K
β = 95.79 (3)°	Block, colorless
γ = 99.68 (3)°	0.22 × 0.20 × 0.18 mm
*V* = 570.9 (2) Å^3^	

### Data collection

**Table:**

Bruker SMART CCD diffractometer	2597 independent reflections
Radiation source: fine-focus sealed tube	1839 reflections with *I* > 2σ(*I*)
graphite	*R*_int_ = 0.016
φ and ω scans	θ_max_ = 27.5°, θ_min_ = 3.5°
Absorption correction: multi-scan (*SADABS*; Sheldrick, 1996)	*h* = −7→6
*T*_min_ = 0.491, *T*_max_ = 0.728	*k* = −13→13
5650 measured reflections	*l* = −14→14

### Refinement

**Table:**

Refinement on *F*^2^	Secondary atom site location: difference Fourier map
Least-squares matrix: full	Hydrogen site location: inferred from neighbouring sites
*R*[*F*^2^ > 2σ(*F*^2^)] = 0.047	H atoms treated by a mixture of independent and constrained refinement
*wR*(*F*^2^) = 0.180	*w* = 1/[σ^2^(*F*_o_^2^) + (0.1217*P*)^2^ + 0.0113*P*] where *P* = (*F*_o_^2^ + 2*F*_c_^2^)/3
*S* = 1.08	(Δ/σ)_max_ = 0.003
2597 reflections	Δρ_max_ = 0.23 e Å^−^^3^
166 parameters	Δρ_min_ = −0.23 e Å^−^^3^
0 restraints	Extinction correction: *SHELXL97* (Sheldrick, 2008), Fc^*^=kFc[1+0.001xFc^2^λ^3^/sin(2θ)]^-1/4^
Primary atom site location: structure-invariant direct methods	Extinction coefficient: 0.043 (14)

### Special details

**Table:**

Geometry. All e.s.d.'s (except the e.s.d. in the dihedral angle between two l.s. planes) are estimated using the full covariance matrix. The cell e.s.d.'s are taken into account individually in the estimation of e.s.d.'s in distances, angles and torsion angles; correlations between e.s.d.'s in cell parameters are only used when they are defined by crystal symmetry. An approximate (isotropic) treatment of cell e.s.d.'s is used for estimating e.s.d.'s involving l.s. planes.
Refinement. Refinement of *F*^2^ against ALL reflections. The weighted *R*-factor *wR* and goodness of fit *S* are based on *F*^2^, conventional *R*-factors *R* are based on *F*, with *F* set to zero for negative *F*^2^. The threshold expression of *F*^2^ > σ(*F*^2^) is used only for calculating *R*-factors(gt) *etc*. and is not relevant to the choice of reflections for refinement. *R*-factors based on *F*^2^ are statistically about twice as large as those based on *F*, and *R*- factors based on ALL data will be even larger.

### Fractional atomic coordinates and isotropic or equivalent isotropic displacement parameters (Å^2^)

**Table:**

	*x*	*y*	*z*	*U*_iso_*/*U*_eq_	
O3	0.4674 (2)	0.34070 (13)	0.74631 (11)	0.0681 (4)	
O1	0.2984 (2)	0.09910 (13)	1.08787 (10)	0.0676 (4)	
O2	0.52301 (18)	0.24189 (11)	1.00894 (9)	0.0549 (3)	
N1	0.1757 (2)	0.16596 (12)	0.81259 (10)	0.0476 (3)	
N2	0.1444 (2)	0.10360 (14)	0.89965 (11)	0.0536 (3)	
C1	0.0453 (2)	0.20676 (14)	0.63011 (12)	0.0453 (3)	
C9	0.3238 (3)	0.14591 (16)	1.00535 (13)	0.0505 (4)	
C7	−0.0055 (2)	0.13722 (14)	0.71973 (12)	0.0440 (3)	
C3	0.3121 (3)	0.36489 (19)	0.55821 (16)	0.0682 (5)	
H3B	0.4653	0.4273	0.5690	0.082*	
C2	0.2752 (3)	0.30312 (15)	0.64688 (14)	0.0512 (4)	
C10	0.7203 (3)	0.29608 (19)	1.12246 (15)	0.0635 (4)	
H10A	0.7912	0.2169	1.1274	0.076*	
H10B	0.6526	0.3396	1.2002	0.076*	
C8	−0.2561 (3)	0.0382 (2)	0.69869 (19)	0.0638 (5)	
C5	−0.0996 (4)	0.2415 (2)	0.43765 (17)	0.0728 (5)	
H5A	−0.2254	0.2202	0.3677	0.087*	
C6	−0.1370 (3)	0.17999 (19)	0.52442 (16)	0.0626 (4)	
H6A	−0.2912	0.1176	0.5120	0.075*	
C4	0.1279 (4)	0.3354 (2)	0.45604 (17)	0.0737 (5)	
H4A	0.1555	0.3787	0.3987	0.088*	
C11	0.9167 (3)	0.40714 (19)	1.10931 (17)	0.0706 (5)	
H11A	1.0503	0.4458	1.1829	0.106*	
H11B	0.8441	0.4846	1.1043	0.106*	
H11C	0.9825	0.3626	1.0323	0.106*	
H3A	0.407 (5)	0.283 (3)	0.794 (2)	0.107 (8)*	
H2A	−0.001 (4)	0.039 (2)	0.8940 (17)	0.074 (5)*	
H8A	−0.392 (5)	0.072 (3)	0.670 (2)	0.127 (9)*	
H8B	−0.298 (5)	0.034 (3)	0.767 (3)	0.133 (10)*	
H8C	−0.259 (6)	−0.042 (4)	0.637 (3)	0.139 (11)*	

### Atomic displacement parameters (Å^2^)

**Table:**

	*U*^11^	*U*^22^	*U*^33^	*U*^12^	*U*^13^	*U*^23^
O3	0.0548 (6)	0.0760 (7)	0.0715 (7)	−0.0132 (5)	−0.0027 (5)	0.0428 (6)
O1	0.0639 (7)	0.0847 (8)	0.0621 (6)	−0.0099 (5)	−0.0004 (5)	0.0522 (6)
O2	0.0530 (6)	0.0615 (6)	0.0509 (5)	−0.0086 (4)	−0.0006 (4)	0.0349 (5)
N1	0.0485 (6)	0.0524 (6)	0.0464 (6)	0.0009 (4)	0.0051 (5)	0.0302 (5)
N2	0.0497 (7)	0.0614 (7)	0.0534 (6)	−0.0050 (5)	0.0018 (5)	0.0365 (5)
C1	0.0476 (7)	0.0474 (7)	0.0444 (7)	0.0075 (5)	0.0079 (5)	0.0239 (5)
C9	0.0533 (7)	0.0528 (7)	0.0499 (7)	0.0019 (6)	0.0069 (6)	0.0306 (6)
C7	0.0426 (6)	0.0461 (7)	0.0435 (6)	0.0042 (5)	0.0072 (5)	0.0209 (5)
C3	0.0708 (10)	0.0677 (10)	0.0746 (10)	−0.0033 (7)	0.0139 (8)	0.0458 (8)
C2	0.0524 (8)	0.0489 (7)	0.0534 (7)	0.0032 (5)	0.0080 (6)	0.0257 (6)
C10	0.0605 (9)	0.0695 (10)	0.0554 (8)	−0.0072 (7)	−0.0040 (7)	0.0326 (7)
C8	0.0498 (8)	0.0802 (11)	0.0650 (9)	−0.0084 (7)	0.0004 (7)	0.0454 (9)
C5	0.0723 (11)	0.0932 (13)	0.0665 (9)	0.0105 (9)	0.0020 (8)	0.0531 (9)
C6	0.0535 (8)	0.0794 (10)	0.0601 (8)	0.0011 (7)	0.0020 (7)	0.0417 (8)
C4	0.0878 (12)	0.0808 (11)	0.0724 (10)	0.0118 (9)	0.0155 (9)	0.0552 (9)
C11	0.0631 (9)	0.0640 (10)	0.0740 (10)	−0.0072 (7)	0.0042 (8)	0.0274 (8)

### Geometric parameters (Å, °)

**Table:**

O3—C2	1.3543 (19)	C3—H3B	0.9300
O3—H3A	0.98 (2)	C10—C11	1.489 (2)
O1—C9	1.2173 (16)	C10—H10A	0.9700
O2—C9	1.3237 (17)	C10—H10B	0.9700
O2—C10	1.4563 (18)	C8—H8A	0.95 (3)
N1—C7	1.2836 (18)	C8—H8B	0.85 (3)
N1—N2	1.3790 (15)	C8—H8C	0.84 (3)
N2—C9	1.3521 (19)	C5—C6	1.374 (2)
N2—H2A	0.92 (2)	C5—C4	1.381 (3)
C1—C6	1.393 (2)	C5—H5A	0.9300
C1—C2	1.408 (2)	C6—H6A	0.9300
C1—C7	1.4737 (18)	C4—H4A	0.9300
C7—C8	1.4988 (19)	C11—H11A	0.9600
C3—C4	1.365 (2)	C11—H11B	0.9600
C3—C2	1.395 (2)	C11—H11C	0.9600
			
C2—O3—H3A	104.2 (15)	O2—C10—H10B	110.3
C9—O2—C10	116.29 (10)	C11—C10—H10B	110.3
C7—N1—N2	119.65 (11)	H10A—C10—H10B	108.6
C9—N2—N1	119.71 (11)	C7—C8—H8A	112.7 (16)
C9—N2—H2A	116.8 (11)	C7—C8—H8B	114 (2)
N1—N2—H2A	123.3 (11)	H8A—C8—H8B	101 (2)
C6—C1—C2	116.97 (12)	C7—C8—H8C	107 (2)
C6—C1—C7	120.60 (12)	H8A—C8—H8C	105 (3)
C2—C1—C7	122.43 (12)	H8B—C8—H8C	117 (3)
O1—C9—O2	124.75 (13)	C6—C5—C4	118.87 (16)
O1—C9—N2	121.90 (13)	C6—C5—H5A	120.6
O2—C9—N2	113.34 (11)	C4—C5—H5A	120.6
N1—C7—C1	116.09 (11)	C5—C6—C1	123.05 (15)
N1—C7—C8	123.84 (12)	C5—C6—H6A	118.5
C1—C7—C8	120.06 (12)	C1—C6—H6A	118.5
C4—C3—C2	121.27 (14)	C3—C4—C5	120.15 (14)
C4—C3—H3B	119.4	C3—C4—H4A	119.9
C2—C3—H3B	119.4	C5—C4—H4A	119.9
O3—C2—C3	117.02 (13)	C10—C11—H11A	109.5
O3—C2—C1	123.31 (12)	C10—C11—H11B	109.5
C3—C2—C1	119.67 (13)	H11A—C11—H11B	109.5
O2—C10—C11	107.04 (13)	C10—C11—H11C	109.5
O2—C10—H10A	110.3	H11A—C11—H11C	109.5
C11—C10—H10A	110.3	H11B—C11—H11C	109.5

### Hydrogen-bond geometry (Å, °)

**Table:**

*D*—H···*A*	*D*—H	H···*A*	*D*···*A*	*D*—H···*A*
N2—H2A···O1^i^	0.92 (2)	2.05 (2)	2.9649 (19)	170.4 (16)
O3—H3A···N1	0.98 (3)	1.68 (3)	2.5728 (19)	149 (2)

Symmetry codes: (i) −*x*, −*y*, −*z*+2.
